# Machine learning model identifies aggressive acute pancreatitis within 48 h of admission: a large retrospective study

**DOI:** 10.1186/s12911-022-02066-3

**Published:** 2022-11-29

**Authors:** Lei Yuan, Mengyao Ji, Shuo Wang, Xinyu Wen, Pingxiao Huang, Lei Shen, Jun Xu

**Affiliations:** 1 School of Automation, Nanjing University of Information Science and Technology, Nanjing, China; 2 Department of Information Center, Wuhan University Renmin Hospital, Wuhan, Hubei China; 3 Department of Gastroenterology, Wuhan University Renmin Hospital, Wuhan, Hubei China; 4 Department of Gastroenterology, The Central Hospital of Wuhan, Tongji Medical College, Huazhong University of Science and Technology, Wuhan, Hubei China; 5 Institute for AI in Medicine, School of Artificial Intelligence, Nanjing University of Information Science and Technology, NanJing, China

**Keywords:** Acute pancreatitis, Intensive care unit, Xgboost, Machine learning

## Abstract

**Background:**

Acute pancreatitis (AP) with critical illness is linked to increased morbidity and mortality. Current risk scores to identify high-risk AP patients have certain limitations.

**Objective:**

To develop and validate a machine learning tool within 48 h after admission for predicting which patients with AP will develop critical illness based on ubiquitously available clinical, laboratory, and radiologic variables.

**Methods:**

5460 AP patients were enrolled. Clinical, laboratory, and imaging variables were collected within 48 h after hospital admission. Least Absolute Shrinkage Selection Operator with bootstrap method was employed to select the most informative variables. Five different machine learning models were constructed to predictive likelihood of critical illness, and the optimal model (APCU) was selected. External cohort was used to validate APCU. APCU and other risk scores were compared using multivariate analysis. Models were evaluated by area under the curve (AUC). The decision curve analysis was employed to evaluate the standardized net benefit.

**Results:**

Xgboost was constructed and selected as APCU, involving age, comorbid disease, mental status, pulmonary infiltrates, procalcitonin (PCT), neutrophil percentage (Neu%), ALT/AST, ratio of albumin and globulin, cholinesterase, Urea, Glu, AST and serum total cholesterol. The APCU performed excellently in discriminating AP risk in internal cohort (AUC = 0.95) and external cohort (AUC = 0.873). The APCU was significant for biliogenic AP (OR = 4.25 [2.08–8.72], *P* < 0.001), alcoholic AP (OR = 3.60 [1.67–7.72], *P* = 0.001), hyperlipidemic AP (OR = 2.63 [1.28–5.37], *P* = 0.008) and tumor AP (OR = 4.57 [2.14–9.72], *P* < 0.001). APCU yielded the highest clinical net benefit, comparatively.

**Conclusion:**

Machine learning tool based on ubiquitously available clinical variables accurately predicts the development of AP, optimizing the management of AP.

**Supplementary Information:**

The online version contains supplementary material available at 10.1186/s12911-022-02066-3.

## Introduction

Acute pancreatitis (AP) is an inflammatory disease of the pancreas, which is the leading cause of admission to hospital for gastrointestinal disorders in the USA and many other countries. Approximately 15–25% AP patients develop moderately severe or severe AP (SAP), and nearly 25% AP patients had to be admitted to an intensive care unit (ICU) with severe complications [1]. Between 1988 and 2003, mortality from AP decreased from 12 to 2%, according to a large epidemiologic study from the United States [2]. However, mortality rates remain much higher among critical patients. A recent Japanese study showed that the mortality rate for SAP is about 16.7% [3]. Mortality of SAP can be decreased with early identifying and individualized precision treatment. Previous studies have shown that precision treatment within 48 h of admission can substantially decrease the mortality rate of SAP [4]. As a consequence, to identify these patients at admission and at 48 h post-admission and offer targeted therapeutic approaches, we develop a new and more accurate scoring system.

Multiple predictive models have been developed to predict the severity of AP based on clinical, laboratory, and radiological risk factors, various severity grading systems, and serum markers. However, the low specificity (i.e., high false positive rate) of these predictive models, which is complex and cumbersome to complete, combined with the low prevalence of severe AP, led to a low positive predictive value. Especially, many scoring systems (e.g., RANSON, Glasgow) take 48 h to complete, can be used only once, which results in certain limitations [5]. Recently, it is confirmed that BISAP score is an accurate means for risk stratification in patients with AP, but its prognostic accuracy is similar to that of the other scoring system [6]. Which means the current predictors have reached a saturation point from the recent and previous data on severity prediction of AP [7]. Meanwhile, none of the scoring systems combined imaging findings with clinical indicators, which could be the reason there was no improvement in the accuracy. Our scoring system innovatively includes both clinical indicators and radiologic markers that are easy to repeatedly access at admission and at 48 h post-admission.

It has been shown that machine learning (ML) models could improve risk prediction in various diseases [8–14], and drug-drug interactions [15, 16]. Results indicate that ML models have advantages compared to conventional logistic or linear regression by considering high-order, non-linear interactions, yielding more stable predictions. Similarly, this model can also be used for predicting the clinic course of AP. Previous studies used ML models to promote the accuracy of predicting AP by combining APACHE II score and C-reactive protein (CRP) [17]. However, there is’t one ML model which combines imaging findings and clinical indicators to predict can be reported. Moreover, these risk scores only focus one import clinic outcome, such as organ failure, sepsis, in-hospital morality and so on, as endpoints, cannot screen the high-risk AP patients to the largest maximum.

Therefore, we aimed to develop and validate a machine learning model (APCU) that incorporated both the radiological signature and clinical risk factors to improve the accuracy of predicting the development of AP in the first 48 h post-admission, and the high mortality in critical ill patients could be reduced.

## Patients and methods

### Patients

**Fig. 1:**
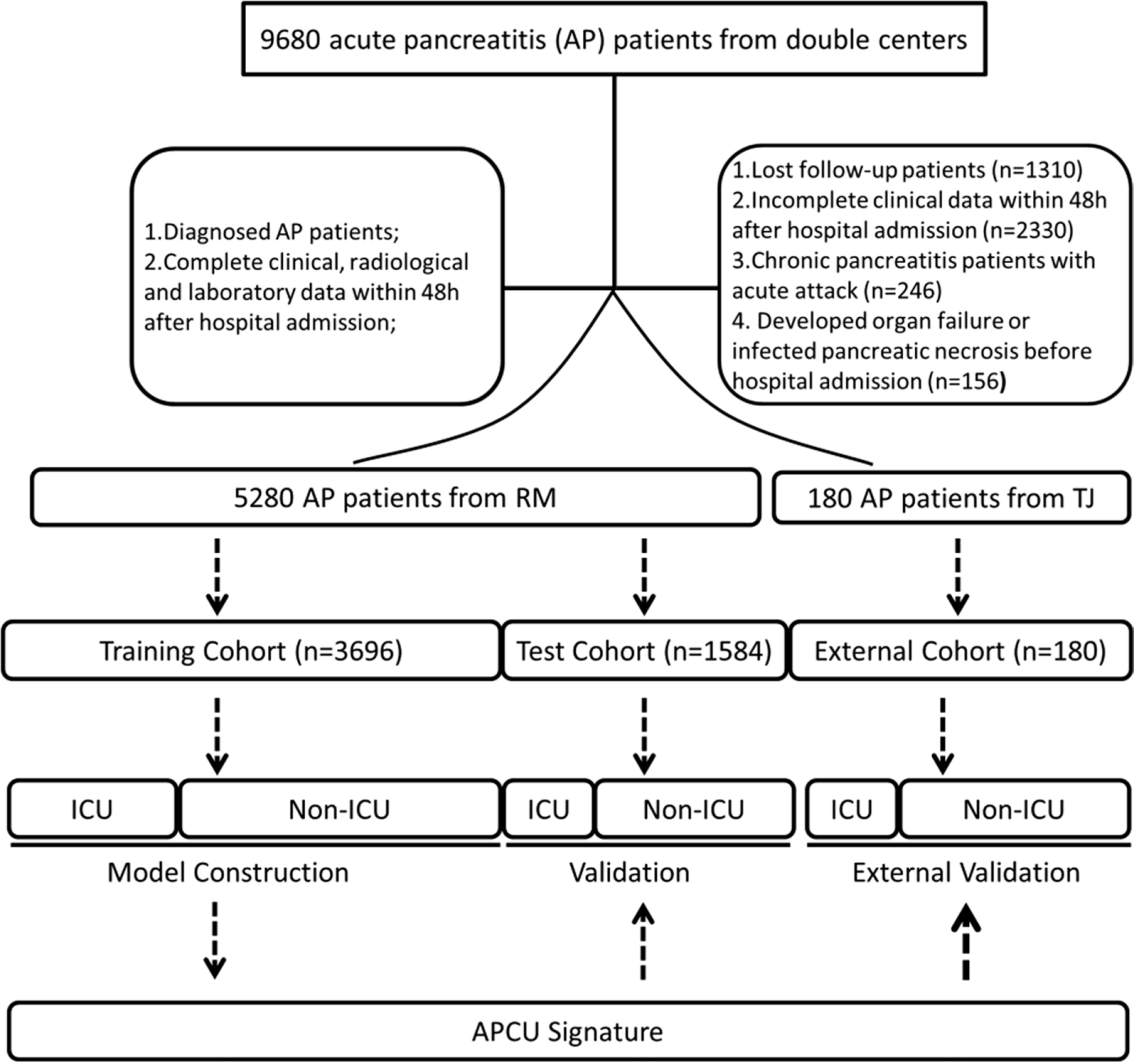
The pipeline of patient selection

Data on consecutive patients who had AP were retrospectively collected from the Renmin Hospital of Wuhan University (RM) between January 6, 2016, and October 22, 2020, and from the Central Hospital of Wuhan, Tongji Medical College, Huazhong University of Science and Technology (TJ) between 2018 and 2019. The study was approved by the Institutional Ethics Committee of the Renmin Hospital of Wuhan University (2021-RM-02106) and the Central Hospital of Wuhan (2021ks06109). Informed consent was waived from all patients for their data to be used for research. The methods and reporting of results adhering to Transparent Reporting of a multivariable prediction model for Individual Prognosis or Diagnosis (TRIPOD) guidelines: Explanation and Elaboration guidelines [18, 19]. Inclusion criteria: (1) Patients admitted to the hospital with a diagnosis of AP by using international consensus [2]; (2) Patients who admitted for the first occurrence of AP; (3) Patients with complete clinical, radiological and laboratory findings within 48 h after admission; (4) Patients with complete clinical course record. Exclusion criteria: (1) Chronic pancreatitis patients with recurrent acute attacks; (2) Patients who have lost follow-up; (3) incomplete clinical data within the first 48 h after admission; (4) pancreatitis cancer; (5) AP caused by endoscopic surgery, developed organ failure, infected pancreatic necrosis or both before hospital admission. Organ failure is defined as a score of two or more for any one of three organ systems (respiratory, cardiovascular, or renal) using the modified Marshall scoring system [20]. Patients were stratified into high-risk or low-risk groups based on the likelihood who will suffer from critical illness or not. The workflow of patient selection is illustrated in Fig. 1.

### Endpoints

We defined the admission of ICU as the endpoint of the follow-up. Patient admission to the ICU was at the discretion of the medical or surgical team based on physiologic variables, laboratory criteria according to the guidelines for ICU admission, discharge, and triage issued by the American College of Critical Care Medicine [21] and the Revised Atlanta Classification of Acute Pancreatitis [22]. The ICU admission criteria included: (1) moderate AP patients with transient organ failure or local or systemic complications; (2) systemic complications without persistent organ failure (< 48 h); (3) pancreatic and peripancreatic abscesses; (4) digestive tract fistula; (5) systemic infection; (6) intra-abdominal hypertension; (7) abdominal compartment syndrome; (8)pancreatic encephalopathy; (9)sepsis; (10)moderate SAP; (11) SAP or critical AP patients, including persistent one or multiple organ failure, infected pancreatic necrosis, or both.

### Potential predictive variables

Clinical variables associated with intensive care unit risk were assessed a priori based on clinical importance, scientific knowledge, and predictors identified in previously literatures [23–26]. Variables with more than 20% missing values were excluded in our study. A total of 59 variables were collected as potential predictive factors, including sex, age, temperature, heart rate, systolic blood pressure, diastolic blood pressure, mental status, BMI, pathogenesis, alcohol, comorbid diseases, pleural effusions, pulmonary infiltration, Epidermal Growth Factor Receptor (eGFR), Urea/Serum Creatinine (Ur/Cr), Total Protein (TP), Total Bilirubin (TBIL), Serum Total Cholesterol (TC), Direct Bilirubin (DBIL), Anion Gap (AG), Aspartate Amino Transferase (AST), Triglyceride (TG), Globulin (GLB), Prealbumin (PA), Glucose (Glu), Uric Acid (UA), Urea, Serum Sodium (Na), Serum Magnesium (Mg), Serum Chlorine (Cl), Serum Phosphate (IP), Alkaline Phosphatase (ALP), Serum Potassium (K), Serum Creatinine (Cr), Serum Calcium (Ca), Total Carbon Dioxide (TCO2), Cholinesterase (CHE), Alanine Aminotransferase (ALT), Albumin (ALB), ratio of albumin and globulin (A/G), γ-glutamyl transpeptidase (GGT), ALT/AST, Neutrophil (Neu), Percentage of Neutrophilic Granulocyte (Neu%), Mean Platelet Volume (MPV), Platelet (PLT), Platelet Volume (PLV), Hemoglobin (Hb), Lymphocyte (LYM), Percentage of Lymphocyte (LYM%), Procalcitonin (PCT), Mean Corpuscular Volume (MCV), Hematocrit (HCT), Red Blood Cell (RBC), Percentage of Monocytes (Mono%), White Blood Cell (WBC), CRP, Serum Lipase (LIPA) and Serum Amylase (AMY). The laboratory indicators, abbreviations, and normal ranges are summarized in Additional file 1: Table S2. And all data used for analysis were the first examination results within the first 48 h after admission. Imputation for missing variables was taken into consideration if the missing values were less than 20%. And the missing data were imputed using R package ‘DMwR’.

### Feature selection

The least absolute shrinkage and selection operator (LASSO) on the logistic regression model with bootstrap method was employed to select the most important variables for constructing prediction models [27], compared with minimum redundancy maximum relevance (MRMR) and Boruta feature selection methods. L1-penalized absolute shrinkage with 20-fold cross validation was conducted for LASSO variable selection process. The most predictive variables with the minimum λ were reported using R package ‘glmnet’. Notable, λ is the optional user-supplied lambda sequence and glmnet chooses its own sequence, aiming to get better convergence. AP risk score was constructed using the coefficients of statistically significant variables weighted by the multivariable logistic regression model in the training cohort. Backward stepwise selection with Akaike’s information criterion was applied to select statistically significant factors for the multivariable logistic regression model; the *P* value threshold was 0.05 (*P* < 0.05) for including the significant variables from the analysis.

### Models construction

The whole cohort was split into 70% training and 30% validation sets. This was to optimize the tradeoff between the robustness of the training sample and the number of events in the test set. Training cohort was used to build prediction models with fivefold cross-validation, whereas the validation cohort was used to validate the models performance. In the training cohort, five machine learning models, including support vector machines with linear kernel (SVM-linear), support vector machines with sigmoid kernel (SVM-sigmoid), support vector machines with radial basis kernel (SVM-radial), logistic regression and xgboost [28], were constructed, using variables identified by LASSO regression analysis. We follow the TRIPOD guidelines [18, 19] to construct the prediction models using identified variables by LASSO. The R packages ‘e1071’, ‘glmnet’ and ‘xgboost’ were employed to build SVM-linear, SVM-sigmoid, SVM-radial, logistic regression and xgboost models, respectively. The Hosmer–Lemeshow test was used to test the goodness of fit for the constructive models.

### Xgboost algorithm

eXtreme Gradient Boosting (Xgboost) is a machine learning technique with gradient boosting method that combines the regression tree [28]. Xgboost has been widely recognized in the machine learning literature [29–31], data mining challenges and disease outcome prediction. By adjusting the hyper-parameters, the xgboost could assemble weak prediction models to an optimal and accurate classifier, with the most predictive features. Additionally, the xgboost could handle missing clinical values effectively, which is common in live clinical work [14].

### Models assessment

The models performances were evaluated by the predictive accuracy (ACC) for individual outcomes (discriminating ability), sensitivity (SEN), specificity (SPC), and the area under the curve (AUC). The Youden index (i.e., sensitivity + specificity − 1) was used to identify the optimal cutoff value in the training cohort and validation cohort, as the equal importance of sensitivity and specificity for AP. The patients will be stratified into high-risk group and low-risk group based on the best cut-off value. We also used the AUC, sensitivity and specificity to compare the accuracy of different types of models and risk scores (i.e., RANSON, SIRS). DeLong test was used to compare AUCs of different models.

The decision curve analysis was employed to evaluate the standardized net benefit of the probability threshold used to categorize observations as 'high risk. The decision curve analysis incorporates consequences and therefore informs the decision of whether to use a model at all, or which of several models is optimal [32]. In the decision curve, the x-axis represents the threshold probability, and the y-axis measures the net benefit. The net benefit was calculated by summing the benefits (true-positive results) and subtracting the harms (false-positive results), weighted by the relative harm of a false-positive and false-negative result. The R package ‘rmda’ was employed to conduct the decision curve analysis.

### Statistical analysis

Continuous variables are reported as mean (SD) or medians with interquartile ranges (IQRs) for skewed distributed variables and compared using an unpaired, 2-tailed *t*-test or Mann–Whitney *U* test. Categorical variables were reported as whole numbers and proportions (n [%]) and compared using the χ^2^ test or Fisher exact test. Shapiro–Wilk normality test was performed to compute the data normality. Imputation for missing variables was taken into consideration if the missing values were less than 20%. The k-nearest neighbors were used to fill in the unknown (NA) values. For NA value, it will impute for k most similar cases and use the values of these cases to fill in the unknowns. The NA values were filled using R package ‘DMwR’. Continuous predictors (i.e., age [33], obesity [26]) were categorized according to the previous researches before analyzing, APACHE II [33], RASON [34], SIRS [35] and NEWS [36] were used as categorical variable. Different types of risk scores were compared using multivariate analysis and visualized with a forestplot, using R package ‘forestplot’.

In all data analyses, *P* < 0.05 was considered statistically significant. Odds ratios (ORs) were reported with their 95% confidence intervals (95% CIs) to evaluate the effect size of important clinical factors. All analyses were performed using R software (version 4.0.4, http://www.r-project.org).

## Results

### Study population

A total of 5280 patients with AP were enrolled in the internal cohort (D_internal_). For D_internal_, 156 (59.1%) were men; more than 50% of the patients were less than 50 years old. Nearly 20% patients had pulmonary infiltrates (16.3%) within 48 h after hospital admission. About 30% patients had comorbid disease. At the end of follow-up endpoint, approximately 15% patients had ICU involvement. Characteristics of the training and test sets were presented in Table 1. No statistically significant were observed between training and test sets (*P* > 0.05).

**Table 1:** Demographics and clinical characteristics of D_internal_

Variables	Total (n = 5280)	Test (n = 1584)	Training (n = 3696)	*P*
Age, n (%)				
< 50	2880 (54.6)	931 (58.8)	1949 (52.7)	0.655
< 75	1940 (36.7)	535 (33.8)	1406 (38.0)	
≥ 75	460 (8.7)	118 (7.5)	341 (9.3)	
Mental status, n (%)				
Awake	4120 (78.0)	1129 (71.3)	2991 (80.9)	0.276
Somnolence	460 (8.7)	198 (12.5)	262 (7.1)	
Stupor	480 (9.1)	158 (10.0)	322 (8.7)	
Coma	220 (4.2)	99 (6.2)	121 (3.3)	
Comorbid diseases, n (%)				
Yes	1563 (29.6)	396 (25.0)	1167 (31.5)	0.286
No	3717 (70.4)	1188 (75.0)	2532 (68.5)	
Pulmonary infiltration, n (%)				
Yes	861 (16.3)	336 (21.2)	525 (14.2)	0.255
No	4419 (83.7)	1248 (78.8)	3171 (85.8)	
TC, median [IQR]	4.20 [3.4, 5.2]	4.14 [3.5, 5.0]	4.25 [3.4, 5.2]	0.810
AST, median [IQR]	25.0 [18.0, 42.0]	24.00 [17.4, 50.0]	25.00 [18.0, 39.0]	0.791
Glu, median [IQR]	7.60 [5.3, 10.1]	8.54 [5.5, 11.6]	7.12 [5.3, 9.9]	0.105
Urea, median [IQR]	5.08 [3.7, 6.8]	4.79 [3.6, 6.8]	5.26 [3.9, 6.7]	0.404
CHE, mean (SD)	8503.59 (3060.8)	8061.50 (3288.8)	8695.81 (2935.5)	0.123
A/G, mean (SD)	1.66 (0.3)	1.61 (0.3)	1.68 (0.3)	0.140
ALT/AST, median [IQR]	0.86 [0.5, 1.2]	0.83 [0.5, 1.2]	0.86 [0.6, 1.2]	0.317
Neu%, median [IQR]	82.25 [72.9, 88.3]	82.9 [73.6, 88.4]	82.25 [72.0, 88.2]	0.547
PCT, median [IQR]	0.34 [0.1, 1.0]	0.44 [0.2, 1.2]	0.30 [0.1, 0.8]	0.034
ICU, n (%)				
Yes	4499 (85.2)	1299 (82.0)	3200 (86.6)	0.164
No	781 (14.8)	285 (18.0)	496 (13.4)	

Abbreviation and normal range: more details could found in Additional file 1: Table S2

### Discriminative features

**Fig. 2:**
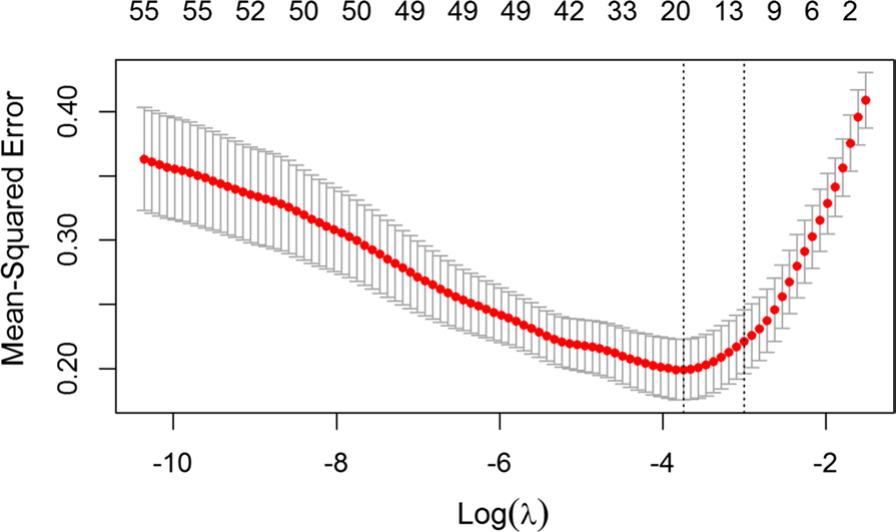
Features selection by Lasso regression. The figure shows the relationship between the log (λ), the number of features in the model, and the mean square error (MSE). λ is the optional user-supplied lambda sequence. Dashed vertical lines were drawn at the optimal values by using the minimum criteria and the 1 standard error of the minimum criteria (the 1-SE criteria). The left dashed line represents the model achieved the minimum MSE with corresponding log(λ) and number of features. The right dashed line represents log(λ) of 1 standard error from MSE with corresponding number of features

LASSO feature selection was used to select the most predictive features, compared with MRMR and Boruta, as best predictive performances (Additional file 1: Table S3). Fifty-nine potential variables measured within 48 h post-hospitalization (Additional file 1: Table S4) were entered into the LASSO regression. 13 variables were selected as informative predictors significantly after LASSO regression selection, including age, comorbid disease, mental status, pulmonary infiltrates, procalcitonin, percentage of neutrophilic granulocytes, ALT/AST, ratio of albumin and globulin (A/G), cholinesterase, urea, glucose, aspartate amino transferase and serum total cholesterol (Fig. 2).

### Internal validation

**Fig. 3:**
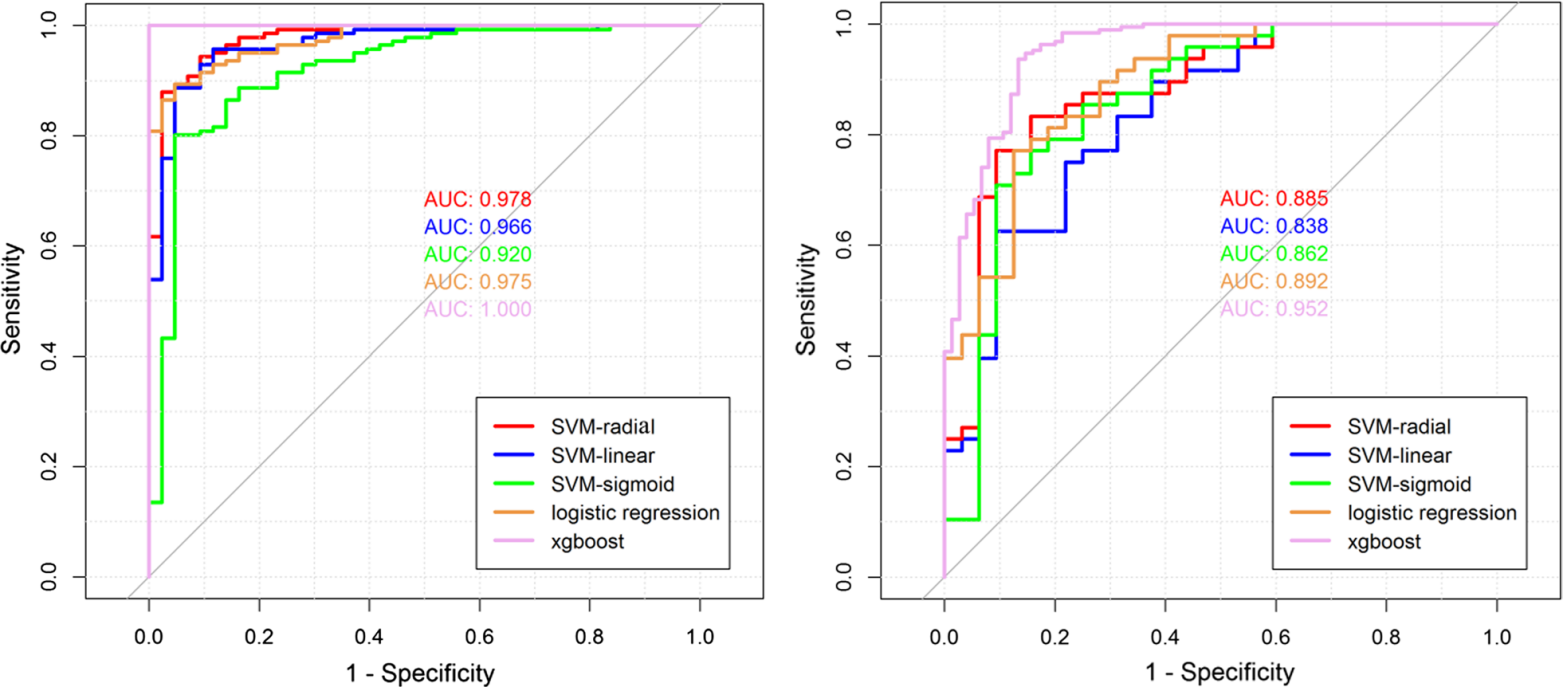
Five different models’ performances (ROC curves) in the training (left) and test (right) cohorts

SVM-linear, SVM-sigmoid, SVM-radial, logistic regression, and xgboost models were constructed using the most informative features identified by LASSO regression selection. The Hosmer Lemeshow test yielded none-statistically significance for SVM-linear (*P* = 0.296), SVM-sigmoid (*P* = 0.452), SVM-radial (*P* = 0.263), logistic regression (*P* = 0.530) and xgboost models (*P* = 0.702), respectively. The xgboost model yielded the highest discriminative performance (ACC = 0.998, SEN = 1.0, SPC = 1.0, AUC = 1.0), compared with the other four models, in the training cohort. Model discrimination results for the prediction of ICU involvement are shown in Additional file 1: Table S5. In the training cohort, all models provided an AUC of greater than 0.90, Fig. 3A. Meanwhile, in the test cohort, the xgboost model can also yielded the best discriminative result with AUC of 0.952, accompanying ACC of 0.863, SEN of 0.889, SPC of 0.792, as shown in Fig. 3B. Therefore, the xgboost model was locked down as the optimal model (APCU) to identity AP patients who are likely to endure critical illness involvement. Patient was stratified into high-risk or low-risk group (threshold = 0.508) based on the best cut-off value determined by Youden index. Namely, a patient will be classified into high-risk group if the probability (APCU output) is more than the threshold.

### Validation on subgroups of AP

**Fig. 4:**
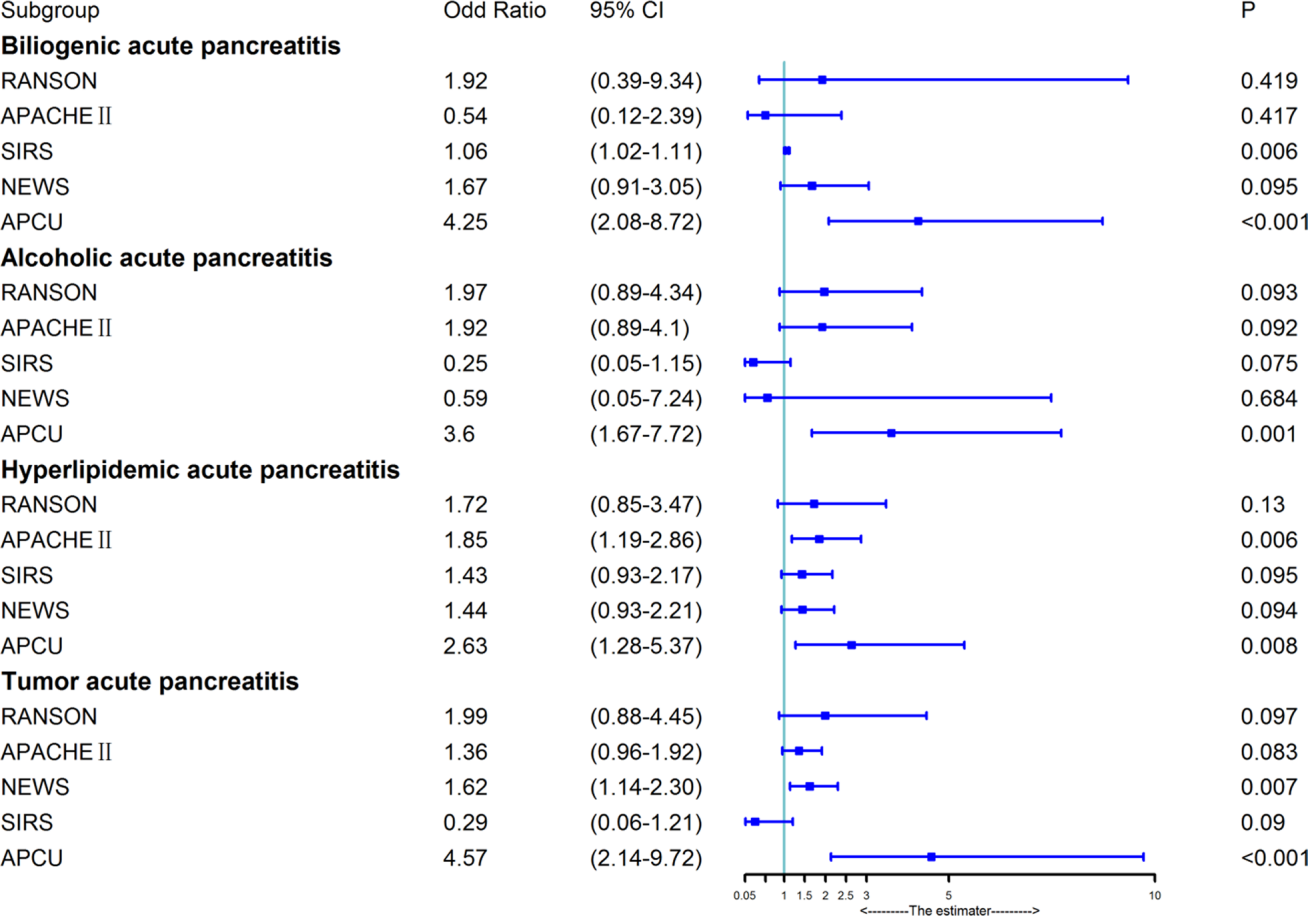
Comparasions of APCU with other risk scores for subgroups of AP

The APCU signature was independently statistically significant for biliogenic AP (OR = 4.25, *P* < 0.001), alcoholic AP (OR = 3.60, *P* = 0.001), hyperlipidemic AP (OR = 2.63, *P* = 0.008) and tumor AP (OR = 4.57, *P* < 0.001), Fig. 4. While the SIRS, APACHE II and NEWS were statistically significant for biliogenic AP (OR = 1.06, *P* = 0.006), hyperlipidemic AP (OR = 1.85, *P* = 0.006) and tumor AP (OR = 1.62, *P* = 0.007), respectively. SIRS has marginal statistically significance for alcoholic AP (OR = 0.25, *P* = 0.075). Together, APCU had highly significantly discriminating ability of ICU involvement in the subgroup of AP, while not for SIRS, APACHE II, or NEWS risk scores.

### External validation

For the external validation cohort (D_external_), a total of 180 AP patients with a mean age of 52 years were enrolled in the independent validation cohort. 32 (17.8%) were eventually developed critical illness (Additional file 1: Table S6). APCU yield an AUC of 0.873, along with the SEN of 0.974 and SPC of 0.750 in the external validation cohort (Additional file 1: Fig. S1).

### Clinical utilization

**Fig. 5:**
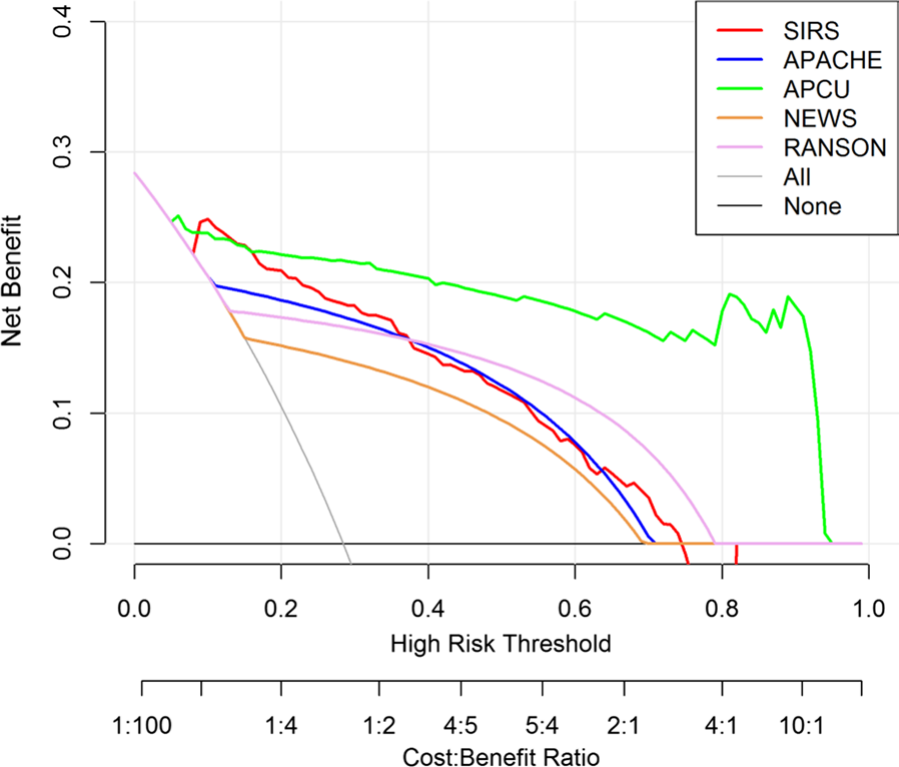
Decision curve analysis for the APCU, APACHE, SIRS, NEWS, and RANSON. The x-axis represented the high-risk threshold, and the y-axis calculated the net benefit (getting benefit from using different prediction models). The pink, green, blue, red, and brown lines represent RANSON, APCU, APACHE, SIRS and NEWS, respectively. The gray line represents the assumption that all patients have ICU involvement. Thin black line represents the assumption that no patients have ICU involvement

The decision curve analysis for the APCU, SIRS, APACHE, NEWS, and RANSON are presented in Fig. 5. The decision curve showed that if the threshold probability of a patient or doctor is 10%, using the APCU to predict ICU admission adds more than 20% net benefit than either the treat-all-patients scheme or the treat-none scheme. Namely, if we choose APCU to predict ICU admission with a 20% probability of diagnosis and treatment, then for every 100 patients using APCU, 23 patients will benefit from using APCU. Comparatively, for every 100 patients using NEWS, 16 patients would benefit from this computer-aided decision, and personalized treatment. When using the APCU to make the decision of whether to undergo personalized treatment, an added clinical net benefit will be achieved than the treat-all scheme or the treat-none scheme. Notable, the APCU model yields the highest clinical net benefit than the other 4 models, comparatively.

## Discussion

In this double-center, retrospective cohort study, we developed and externally validated a novel machine learning tool (APCU) based on clinical, laboratory, and radiologic factors to predict ICU admission in patients with AP. Our results showed that the APCU developed in this study stratified AP patients into high-risk and low-risk groups, showing significantly more discriminative ability than other risk scores (Ranson, APACHE II, SIRS, NEWS) in predicting ICU admission in AP patients and subgroups of AP patients within 48 h after hospital admission (Fig. 4). To our knowledge, this study is the first attempt to use machine learning algorithm to predict ICU admission in AP patients within 48 h post-hospitalization based on ubiquitously available clinical, laboratory, and radiologic findings.

In recent decades, mortality from AP has decreased dramatically. However, mortality rates remain much higher in subgroups of patients with severe disease. By using the APCU, we could identify patients who will undergo intensive surveillance accurately (AUC > 0.90, Fig. 3) and inexpensively. The ability to predict the likelihood of critical illness can help identify patients at increased risk for morbidity and mortality, thereby assisting in appropriate early triage to ICU and selection of patients for specific interventions, as well as reducing the health burden for AP patients.

Early identification of high-risk AP with adverse outcome has been investigated by many researchers for many years. For example, Wu et.al used classification and regression tree (CART) analysis to early predict the in-hospital morality of AP patients with 24 h post-admission [37]. Ranson et al., the first specific risk score system for acute biliary pancreatitis, contains 11 significant prognostic factors for predict severity of AP [38]. Rahul et al. used machine learning (xgboost) to early predict to identify those AP patients who would develop SAP [14], aiming to improve risk stratification of AP patients in clinical settings. Our study is different from the previous researches. We did not set one import clinic outcome as endpoint (i.e., organ failure, sepsis, morality). We distinctly and broadly set ICU admission as the main endpoint, aiming to screen the high-risk AP to the largest maximum. To this end, the APCU was constructed using the clinical, laboratory, and radiologic factors. And the AP patients could get the largest net benefit, comparatively (Fig. 5).

Thirteen clinical factors, including age, comorbid disease, mental status, pulmonary infiltrates, PCT, Neu%, ALT/AST, A/G, CHE, Urea, Glu, AST and TC, were employed to construct the predictive model. Recent literatures have found several of these factors were linked to the development of AP. Frey et al. [39] found that older age is a predictor of a worse prognosis. Kylanpaa et al. [25] declared that procalcitonin is the most rapid general acute-phase reactant at the early stage of AP. Talamini et al. [24] suggest that a pleural effusion and/or pulmonary infiltrate may be associated with necrosis and organ failure in AP patients. Indeed, radiological findings with bilateral pulmonary infiltrates and physiological changes are the most common manifest clinically as acute lung injury (ALI) for AP. Initially, exudative phase with diffuse alveolar damage, microvascular injury, type I pneumocyte necrosis, and influx of inflammatory cells and fluid to the pulmonary interstitium has been witnessed. This make the pulmonary infiltrates as the significant biomarker for the AP, identified as aggressive patients. Our study thereby further complemented these recent findings.

APCU predictions were conducted at the 48 h post-hospitalization. APCU scores were computed using patient radiologic findings, clinical and laboratory results. And the patients were identified as aggressive AP patients were preferentially transferred to ICU. In terms of clinical application, the APCU could be integrated into clinical utilization in several ways. First, it could assist with early triage procedures appropriately and timely. When patients are admitted to hospital, the APUC could infer a predictive score based on the basic history, laboratory, and radiologic findings, which are widely routinely available. This machine learning predictive information could help to prioritize high-risk patients and access to clinical and supportive care, thereby contributing to the optimization of public medical resources.

Another potential application of APCU could help assist physicians with the triage of patients with complicated or rare conditions, especially in areas with scarce medical resources. International Association of Pancreatology (IAP)/American Pancreatic Association (APA) [40], American College of Gastroenterology (ACG) [41], and American Gastroenterological Association guidelines [42] have widely adopted for comprehensive initial assessment, triage and management of AP. Clinicians interpret the clinical history to diagnose, triage and management patients with AP. In this way, a physician could use the machine learning model to help expand his or her differential diagnosis and significantly influence clinician behavior, positively, contributing to broadly improve the management of AP. In addition, a clinical usage formula was provided for easy clinical use and promotion.

Our study has some limitations. Limited sample size was enrolled for constructing the machine learning model and a relatively small sample for internal and independent validation, which potentially limits the generalizability of the model. Next, all enrolled patients were recruited from the limited institutions. Additional independent validation studies are required before this machine learning model could be implemented in clinical workflows.

## Conclusions

Conclusively, this study describes a machine learning framework based on widely routinely available clinical indicators to accurately and earlier predict a patient’s prognosis. The APCU may be useful for optimizing the management of AP. Moreover, this framework could become a triage system for physicians and assist in cases of diagnostic uncertainty or complexity, benefiting the allocation of healthcare resources.

## Supplementary Information

**Additional file 1**. Data repeatability. **Figure S1**. APCU performance in the external cohort. **Table S1**. Parameters setting and description of APCU. **Table S2**. Laboratory indicators abbreviation and normal range. **Table S3**. Evaluation of different combinations for feature selection algorithms and classifiers validation on training set. **Table S4**. Potential predictive variables includes demographics, vitals, radiologic findings and laboratory indicators. **Table S5**. Model discriminative performances in training and test cohort. **Table S6**. Demographics and clinical characteristics of external validation cohort.

## Data Availability

The datasets generated during and/or analysed during the current study are available from the corresponding author on reasonable request.
