# Supplementary material for: Machine learning model identifies aggressive acute pancreatitis within 48 h of admission: a large retrospective study
Source: BMC Med Inform Decis Mak. 2022 Nov 29;22:312. doi: 10.1186/s12911-022-02066-3 (PMC9707001; doi:10.1186/s12911-022-02066-3)
Supplement: Supplementary file 1 — Additional file 1. Data repeatability. Figure S1. APCU performance in the external cohort. Table S1. Parameters setting and description of APCU. Table S2. Laboratory indicators abbreviation and normal range. Table S3. Evaluation of different combinations for feature selection algorithms and classifiers validation on training set. Table S4. Potential predictive variables includes demographics, vitals, radiologic findings and laboratory indicators. Table S5. Model discriminative performances in training and test cohort. Table S6. Demographics and clinical characteristics of external validation cohort. [file 12911_2022_2066_MOESM1_ESM.docx]

Machine learning model identifies aggressive acute pancreatitis within 48h of admission: a large retrospective study

Additional file 1: Data repeatability.

Figure S1: APCU performance in the external cohort.

Table S2: Laboratory indicators abbreviation and normal range.

Table S3. Evaluation of different combinations for feature selection algorithms and classifiers validation on training set

Table S4: Potential Predictive Variables includes demographics, vitals, radiologic findings and laboratory indicators.

Table S5: Model discriminative performances in training and test cohort.

Table S6: Demographics and Clinical Characteristics of external validation cohort.

Additional file 1: Data Repeatability

Xgboost (eXtreme Gradient Boosting package) [[1](#_ENREF_1)] was used to construct the APCU. It is an efficient and scalable implementation of gradient boosting framework by Friedman et al [[1](#_ENREF_1)]. Dummy contrast coding was employed to transform the categorical data to numeric variables. Sparse matrix was used as input parameters to build the xgboost model. xgb.train function was used to get the optimal hyper- parameters from a list of predefined parameters, including nrounds (max number of boosting iterations), colsample_bytree (subsample ratio of columns when constructing each tree), min_child_weight (minimum sum of instance weight needed in a child), eta (learning rate), gamma (minimum loss reduction required to make a further partition on a leaf node of the tree), subsample (subsample ratio of the training instance), max_depth (maximum depth of a tree). A list of control parameters (R package ‘carent’) was employed to control the computational nuances of the train function, including method = ‘CV’ (the resampling method), number =5 (number of resampling iterations). Finally, the optimal hyper-parameters were achieved as following table.

**Table S1. Parameters setting and description of APCU**

| **parameters** | **setting** | **description** |
| --- | --- | --- |
| max_depth | 5 | maximum depth of a tree |
| eta | 0.3 | learning rate |
| gamma | 0.3 | minimum loss reduction required to make a further partition on a leaf node of the tree |
| colsample_bytree | 1 | subsample ratio of columns when constructing each tree |
| min_child_weight | 1 | minimum sum of instance weight needed in a child |
| subsample | 0.5 | subsample ratio of the training instance |
| nround | 35 | max number of boosting iterations |
| objective | binary:logistic | the learning task and the corresponding learning objective |

Additional file 1: Figure S1. APCU performance on the external validation cohort.


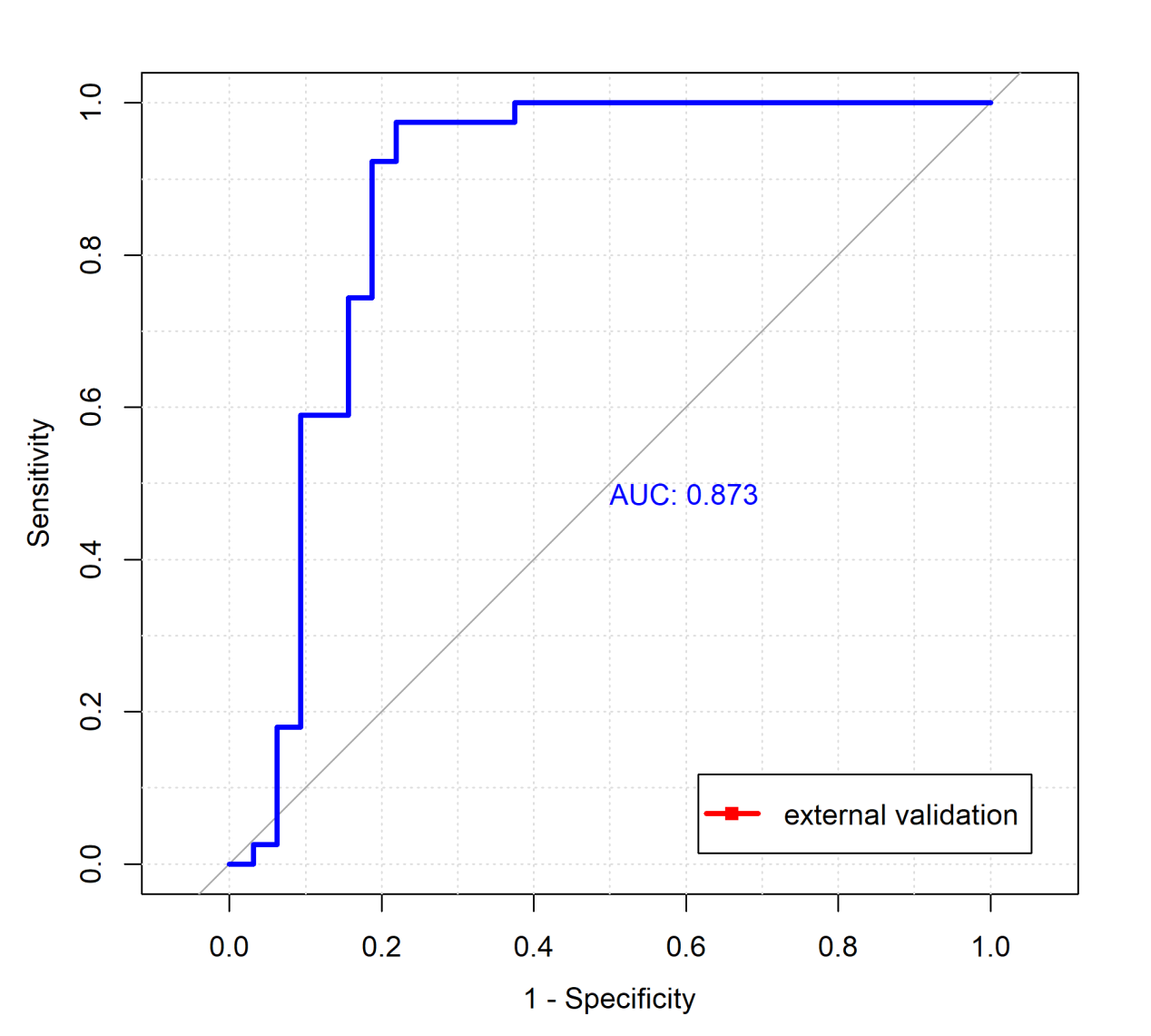


Additional file 1: Table S2. Laboratory indicators abbreviation and normal range.

| Laboratory indicators | Abbreviation | Normal range |
| --- | --- | --- |
| Epidermal Growth Factor Receptor | EGFR | >90 mL/min |
| Urea/ Serum Creatinine | Ur/Cr | -- |
| Total Protein | TP | 65-85 g/L |
| Total Bilirubin | TBIL | 0-23μmol/L |
| Serum Total Cholesterol | TC | <5.2 mmol/L |
| Direct Bilirubin | DBIL | 0-8.0μmol/L |
| Anion Gap | AG | 0-1.0 COI |
| Aspartate Amino Transferase | AST | 13-35 U/L |
| Triglyceride | TG | <1.70 mmol/L |
| Globulin | GLB | 20-40 g/L |
| Prealbumin | PA | 250-400 mg/L |
| Glucose | Glu | 3.9-6.1 mmol/L |
| Uric Acid | UA | 208-428μmol/L |
| Urea | Urea | 3.1-8.0 mmol/L |
| Serum Sodium | Na | 137-147 mmol/L |
| Serum Magnesium | Mg | 0.75-1.02 mmol/L |
| Serum Chlorine | Cl | 99-110 mmol/L |
| Serum Phosphate | IP | 0.85-1.51 mmol/L |
| Alkaline Phosphatase | ALP | 45-125 U/L |
| Serum Potassium | K | 3.5-5.3 mmol/L |
| Serum Creatinine | Cr | 57-111μmol/L |
| Serum Calcium | Ca  TCO2 | 2.11-2.52 mmol/L |
| Total Carbon Dioxide | TCO_2_  TCO2  CHE | 22-33 mmol/L |
| Cholinesterase | CHE | 4900-11900 U/L |
| Alanine Aminotransferase | ALT | 9-50 U/L |
| Albumin | ALB | 40-55 g/L |
| ALB/GLB | A/G | 1.2-2.4 |
| γ-glutamyl transpeptidase | GGT | 10-60 U/L |
| ALT/AST | ALT/AST | -- |
| Neutrophil | Neu | 1.8-6.3 10^9 /L |
| Percentage of Neutrophilic Granulocyte | Neu% | 40-75% |
| Mean Platelet Volume | MPV | 5.5-14.5 fL |
| Platelet | PLT | 125-350 10^9 /L |
| Platelet Volume | PLV | 0.001-0.004 L/L |
| Hemoglobin | Hb | 130-175 g/L |
| Lymphocyte | LYM | 1.1-3.2 10^9 /L |
| Percentage of Lymphocyte | LYM% | 20-50% |
| Procalcitonin | PCT | <0.1 ng/mL |
| Mean Corpuscular Volume | MCV | 82-100 fL |
| Hematocrit | HCT | 0.40-0.50 L/L |
| Red Blood Cell | RBC | 4.3-5.8 10^9 /L |
| Percentage of Monocytes | Mono% | -- |
| White Blood Cell | WBC | 3.5-9.5 10^9 /L |
| C-reactive Protein | CRP | 0-10 mg/L |
| Serum Lipase | LIPA | 23-300 U/L |
| Serum Amylase | AMY | 35-135 U/L |

**Additional file 1: Table S3.** Evaluation of different combinations for feature selection algorithms and classifiers validation on training set

| Dataset | Classifier | Feature selection | AUC | Accuracy | Specificity | Sensitivity |
| --- | --- | --- | --- | --- | --- | --- |
| Training set | SVM-radial | LASSO | 0.978±0.08 | 0.91±0.08 | 0.82±0.05 | 0.67±0.03 |
|  |  | MRMR | 0.67±0.05 | 0.80±0.04 | 0.84±0.02 | 0.69±0.09 |
|  |  | Boruta | 0.76±0.03 | 0.79±0.06 | 0.85±0.02 | 0.62±0.05 |
|  | SVM-linear | LASSO | 0.966±0.02 | 0.74±0.09 | 0.80±0.03 | 0.71±0.08 |
|  |  | MRMR | 0.79±0.06 | 0.77±0.01 | 0.82±0.01 | 0.73±0.01 |
|  |  | Boruta | 0.72±0.06 | 0.87±0.02 | 0.86±0.03 | 0.75±0.03 |
|  | SVM-sigmoid | LASSO | 0.920±0.03 | 0.79±0.06 | 0.82±0.06 | 0.72±0.04 |
|  |  | MRMR | 0.81±0.06 | 0.76±0.04 | 0.80±0.08 | 0.70±0.06 |
|  |  | Boruta | 0.80±0.05 | 0.73±0.08 | 0.79±0.06 | 0.69±0.08 |
|  | Xgboost | LASSO | 1.0±0.03 | 0.998±0.02 | 1.0±0.01 | 1.0±0.08 |
|  |  | MRMR | 0.84±0.02 | 0.88±0.01 | 0.84±0.02 | 0.72±0.04 |
|  |  | Boruta | 0.81±0.05 | 0.84±0.04 | 0.82±0.02 | 0.73±0.07 |
|  | logistic regression | LASSO | 0.975±0.06 | 0.89±0.04 | 0.88±0.03 | 0.78±0.05 |
|  |  | MRMR | 0.81±0.03 | 0.78±0.02 | 0.74±0.05 | 0.82±0.07 |
|  |  | Boruta | 0.79±0.06 | 0.74±0.08 | 0.80±0.06 | 0.77±0.09 |

LASSO: least absolute shrinkage and selection operator; MRMR: minimum redundancy maximum relevance; SVM: [support vector machine](javascript:;).

**Additional file 1: Table S4. Potential Predictive Variables includes demographics, vitals, radiologic findings and laboratory indicators.**

| **Potential predictive variables** | **Details** |
| --- | --- |
| Demographics | age, n(%)， gender, n(%), BMI, n(%), pathogenesis, n(%), alcohol, n(%), comorbid diseases, n(%) |
| Vitals | temperature, mean(SD), heart rate, mean(SD), systolic blood pressure, , mean(SD), diastolic blood pressure, , mean(SD), mental status, n(%) |
| Radiologic findings | Pleural effusions, n(%), Pulmonary infiltration, n(%) |
| Laboratory indicators | eGFR, median[IQR], Ur/Cr, median[IQR], TP ,mean(SD), TBIL, median[IQR], TC, median[IQR], DBIL, median[IQR], AG, mean(SD) ,AST, median[IQR], TG, median[IQR], GLB ,mean(SD), PA ,mean(SD), Glu, median[IQR], UA, median[IQR], Urea, median[IQR], Na, mean(SD), Mg, mean(SD), Cl, mean(SD), IP, mean(SD), ALP, median[IQR], K, mean(SD), Cr, median[IQR], Ca, median[IQR], TCO2, median[IQR], CHE, mean(SD), ALT, median[IQR], ALB, mean(SD), A/G, mean(SD), GGT, median[IQR], ALT/AST, median[IQR], Neu, median[IQR], Neu%, median[IQR], MPV, median[IQR], PLT, mean(SD), PLV, mean(SD), Hb, mean(SD), LYM, median[IQR], LYM%, median[IQR], PCT ,median[IQR], MCV, mean(SD), HCT, mean(SD), RBC, mean(SD), Mono%, median[IQR], WBC, median[IQR], CRP, median[IQR], LIPA, median[IQR], AMY, median[IQR] |

Additional file 1: Table S5: Model discriminative performances in training and test cohort.

| **models** | **Training cohort** | | | | **Test cohort** | | | |
| --- | --- | --- | --- | --- | --- | --- | --- | --- |
|  | ACC | SEN | SPC | AUC | ACC | SEN | SPC | AUC |
| **SVM-linear** | 0.929 | 0.841 | 0.957 | 0.962 | 0.838 | 0.818 | 0.759 | 0.889 |
| **SVM-sigmoid** | 0.875 | 0.794 | 0.893 | 0.919 | 0.713 | 0.833 | 0.786 | 0.861 |
| **SVM-radial** | 0.940 | 0.971 | 0.933 | 0.981 | 0.863 | 0.850 | 0.750 | 0.847 |
| **Logistic regression** | 0.891 | 0.780 | 0.923 | 0.975 | 0.825 | 0.875 | 0.804 | 0.893 |
| **xgboost** | 0.998 | 1.00 | 1.00 | 1.00 | 0.863 | 0.889 | 0.792 | 0.952 |

Additional file 1: Table S6 Demographics and Clinical Characteristics of external validation cohort

| **Variables** | **Overall(N=180)** | **ICU (N=32)** | **Non-ICU (N=148)** | **P** |
| --- | --- | --- | --- | --- |
| age | 51.8 (16.4) | 58.9 (15.8) | 45.9 (14.8) | 0.001 |
| mental status |  |  |  | 0.037 |
| Awake | 132 (73.5%) | 22 (69.4%) | 110 (74.3%) |  |
| Somnolence | 19(10.5%) | 3 (10.6%) | 16 (10.8%) |  |
| Stupor | 18 (9.9%) | 4 (12.5%) | 14 (9.5%) |  |
| Coma | 11(6.1%) | 3 (10.6%) | 8 (5.4%) |  |
| comorbid disease |  |  |  | <0.001 |
| no | 127 (70.8%) | 20 (62.5%) | 107 (71.8%) |  |
| yes | 53 (29.2%) | 12 (37.5%) | 41 (28.2%) |  |
| pulmonary infiltrates |  |  |  | <0.001 |
| no | 150 (63.4%) | 24 (75.0%) | 126 (85.1%) |  |
| yes | 30 (16.7%) | 8 (25.0%) | 22 (14. 9%) |  |
| PCT | 0.92 (1.17) | 1.27 (1.42) | 0.63 (0.82) | 0.031 |
| Neu% | 79.0 (10.4) | 83.6 (7.57) | 75.3 (10.9) | <0.001 |
| ALT/AST | 0.90 (0.46) | 0.66 (0.28) | 1.10 (0.49) | <0.001 |
| A/G | 1.62 (0.35) | 1.45 (0.35) | 1.76 (0.29) | <0.001 |
| CHE | 8428 (3270) | 6773 (3059) | 9786 (2804) | <0.001 |
| Urea | 6.06 (2.88) | 7.33 (3.28) | 5.01 (2.01) | 0.001 |
| Glu | 8.17 (3.43) | 9.90 (3.18) | 6.75 (2.98) | <0.001 |
| AST | 35.9 (25.90) | 45.7 (30.70) | 27.8 (18.00) | 0.005 |
| TC | 4.30 (1.35) | 4.04 (1.18) | 4.52 (1.45) | 0.031 |

**Reference**

1. Friedman JH: **Greedy function approximation: A gradient boosting machine.** *Annals Of Statistics* 2001, **29:**1189-1232.
